# Supplementary material for: Are carbon emissions trading and green financial instruments synergistic? -Comprehensive quantitative research based on content analysis
Source: PLoS One. 2024 Mar 7;19(3):e0298601. doi: 10.1371/journal.pone.0298601 (PMC10919601; doi:10.1371/journal.pone.0298601)
Supplement: S1 Data — (ZIP) [file pone.0298601.s001.zip › Data/地级市Z_F工作报告.zip!/╔╜╬≈/┤≤═1⁄4╩╨/└┤╫╘╣1⁄2╓┌║┼í╛┬φ┐╦╩2╛▌═°í┐_2012.docx]

各位代表:

现在,我代表市人民政府向大会作工作报告,请予审议,并请市政协委员和其他列席人员提出意见。

一、过去四年工作回顾

过去四年,是大同城市面貌发生深刻变化、综合竞争实力全面增长、转型跨越发展积蓄力量和基础的四年。四年来,全市地区生产总值从2007年的517.04亿元增加到2011年的842.59亿元,年均增长10.96%;财政总收入从93.04亿元增加到162.26亿元,年均增长14.92%;一般预算收入从36.71亿元增加到64.64亿元,年均增长15.19%;全社会固定资产投资从214.79亿元增加到664.91亿元,累计完成2172.4亿元,年均增长32.64%;社会消费品零售总额从184.95亿元增加到356.57亿元,年均增长17.83%;城镇居民人均可支配收入从11508元增加到18915元,年均增长13.23%;农民人均纯收入从3034元增加到4936元,年均增长12.94%;万元GDP综合能耗从2.32吨标煤下降到1.42吨标煤,下降38.79%;化学需氧量、二氧化硫超额完成减排任务。

四年发展的成就集中体现在六个“基本形成”:

——园区承载发展的战略布局基本形成,转型跨越迈出坚实步伐。装备制造、新医药、新材料、清洁能源、光伏产业、循环经济、煤化工、冶金管材等占地160平方公里的八大园区,空间集中开发,资源集约利用,产业集群发展,服务集聚配套,成为转型发展的基本平台。中国化工、中国重汽、冀东集团、中国医药、中海油、陕西重汽、庞大、华润、富士康、保利协鑫、中联重科、亿丰、华锐风电、天津建材等一批大集团、大企业落户园区。园区经济成为大同转型跨越发展的基本特征。煤炭资源整合和煤矿兼并重组完成。中国煤炭企业现代化装备水平最高、产能最大的同煤塔山2000万吨和同忻1000万吨矿井竣工投产,东周窑、马道头两个千万吨矿井基本建成,千亿同煤的目标提前实现。全市煤炭产量由7000万吨突破1亿吨,电力装机容量由340万千瓦增加到770万千瓦。

——双城同辉共美的建设蓝图基本形成,特色城市展现美好前景。持续开展大规模的城市建设,全市新建改造城市道路147条310公里、6座跨河大桥。城市改造建设拆迁面积1000多万平方米,城市棚户区和城中村改造取得决定性成果,城市规划建设面积由90平方公里扩展到180平方公里。四年新增建筑面积2000万平方米,新增绿化面积1700万平方米,新增集中供热面积3000万平方米。城市面貌和格局发生翻天覆地的变化。古城墙修复完成四分之三,御东太阳宫、博物馆、图书馆、大剧院、美术馆、体育中心等重大项目建设顺利,6.8平方公里的文瀛湖基本完工,这些具有大同地标性的建筑和景观,积累着量变到质变的历史性嬗变,折射出一轴双城的无限魅力。

——中国古都引领的产业构架基本形成,文化复兴产生深远影响。以中国大古都为龙头,以中国历史文化名城为主体,以世界遗产云冈石窟、国家风景名胜区北岳恒山为两翼,打造名都、名城、名窟、名山四大品牌。名城保护复兴工程进展顺利,云冈大景区建设基本完成,北岳恒山深度开发成效明显。关帝庙、观音堂、浑源文庙、圆觉寺、律吕神祠被列为新的国保单位。华强集团方特欢乐世界、煤气工业遗址文化创意、晋华宫矿井文化体验加快开发建设。文化体制改革取得初步成效。中国国际雕塑双年展等文化会展节庆活动,迸发出深远的影响和潜力。

——四轮联动发展的农业格局基本形成,县域经济步入良性轨道。龙头带动,基地联动,市场拉动,项目驱动,农业产业化发展势头强劲。龙头企业由102户增长到150户,北京新发地、顺鑫、承德露露、江苏雨润、中储粮、荷兰瑞克斯旺等一批国内外知名企业落户大同,华晟果蔬、夏进乳业、四方高科、佳鑫苦荞、东方物华等一批龙头企业快速发展。设施农业由1万亩达到12.8万亩,畜禽标准化养殖小区达到377个,万头以上规模的养猪园区达到13个,千头以上规模的奶牛园区达到17个。孤山水库、唐河水库、引黄入同等重点工程基本完成。农村两轮“五个全覆盖”顺利推进,省级现代农业示范区取得新进展。大呼、荣乌、天大、同源、广源等覆盖灵丘、广灵、浑源、天镇、阳高、左云、大同、新荣等八个县区,全长387公里的5条高速公路基本完成。全长60公里的208国道大同段改扩建工程完成70%。植树造林完成135万亩,森林覆盖率从15%提高到20.11%。县区经济特色优势逐步显现,县域经济实力不断壮大。

——民生问题密切的服务体系基本形成,幸福大同结出丰硕成果。按照“基础设施配套、城市功能完善、生态环境良好、居住品质提升”的标准,完成各类保障房、安置房800万平方米、9.1万套,数万户群众住房条件得到根本改变。就业水平稳定提高。医疗保险实现全覆盖,新型农保八个试点县进展顺利,城乡低保实现应保尽保,社会救助体系进一步完善。投入45亿元解决市属关闭破产企业330户、8.3万下岗职工的安置和善后问题。投资百亿元新建改造几乎遍及全市城乡所有中学、小学共656所,建筑面积256万平方米。投资30亿元,新建市三医院外科楼、市一医院、五医院、六医院、中医院等重大工程,与民生关系密切的教育、卫生服务体系发生历史性深刻变化。“平安大同”建设深入推进,社会治安综合治理进一步加强,群众安全感增强,安全生产保持平稳。

——行政集中审批的改革制度基本形成,政府职能实现初步转变。坚持依法行政,自觉接受人大及其常委会监督,支持政协参政议政,办理人大代表建议521件、政协委员提案1289件,办复率100%。制定政府规章9件,提请人大审议地方性法规11件。认真听取各民主党派、工商联、无党派人士的意见,支持工会、共青团、妇联等人民团体开展工作。大力推行政务公开,加强电子政务建设。深化行政审批制度改革,将市级原有的411项行政审批事项精简压缩到169项,再造流程,创新载体,阳光作业,严格监管,审批法定时限缩短二分之一,政务大厅成为政府为民服务的平台,展示大同形象的窗口。严格各项制度,从源头上预防腐败,政府管理逐步纳入制度化轨道。

所有这些成绩的取得,归功于省委、省政府和市委的正确领导,归功于全市上下广大干部群众和人大代表、政协委员的团结奋斗,归功于各民主党派、工商联、人民团体和各界人士的广泛参与,归功于驻同中央和省级机关、企事业单位、人民解放军、武警官兵的大力支持。在此,我代表市人民政府,向所有关心、支持和参与大同改革发展的同志们、朋友们表示衷心的感谢和崇高的敬意!

我们也清醒地认识到,大同目前仍处于建设和发展的攻坚阶段,影响经济社会发展的主要矛盾没有根本解决,负重前行,风险伴随的坎还没有跨过,历史的惯性还很大,可持续发展的内生动力和制度环境还没有形成;经济增长的压力和转型发展的要求并存,推动前进的力量与困扰发展的矛盾同在,当前面临的宏观经济形势,使各种交织的问题更加复杂;名城保护任务艰巨,拆迁与建设、营销与管理、保护与开发等亟待破解的难题更加突出;社会管理面临严峻挑战,让人民群众长期共享共同创造的社会财富,成为迫切需要解决的重大问题,文明大同建设任重道远,建立全社会认同的价值观尚需努力;服务型政府建设存在差距,公务员队伍的专业化、知识化水平需进一步提高,效率低下,责任心、事业心不强的问题仍然突出,党风廉政建设需进一步加强。对这些问题,我们要引起高度重视,在今后工作中认真加以解决。

二、下届政府奋斗目标和主要任务

我们所处的时代是一个全球化的时代,下届政府任期正与“十二五”实施期相契合。去年政府已经对“十二五”的奋斗目标和主要任务向大会作了全面报告。“十二五”的目标任务就是下届政府的奋斗目标。在这样一个非常重要的历史关头,我们需要在世界的向度上思考大同的问题,同时也要以时代应有的高度审视世界。大同的发展必须与世界接轨,大同制造,国际水平,才能站在时代的前列,在激烈的国际竞争中立于不败之地;才能抢占发展制高点,在转型发展中实现真正的跨越;才能不断创新和前进,在历史的进程中积累财富。历史厚爱大同,在山西获得国家转型综改试验区重大政策机遇的同时,2013年国际太阳能十项全能竞赛又落户大同,给大同以赛事办城市,以赛事抓发展,以赛事促转型的历史机遇。盛世双喜临门。这是继两汉名郡、北魏京华、辽金陪都、明清重镇之后历史赐予大同的又一次复兴的机遇。2300多年来,大同第一次以主人的身份扮演世界赛事主角。我们必须以登高望远的眼光,海纳百川的胸怀,破釜沉舟的决心,敢为人先的气魄,百折不挠的精神,把大同的赛事办成目前国际水平最高的太阳能赛事,让大同在国际舞台上精彩亮相。

我们要以建设新能源示范城市为目标,走出大同转型路。按照煤炭清洁化、能源绿色化、制造新型化、产业集群化、应用规模化的发展战略,集中力量抓好中海油、同煤投资220亿建设40亿立方煤制天然气、保利协鑫投资200亿建设太阳能光伏完整产业链、同煤、天津建材投资100亿建设300万吨建材钢、同煤投资100亿建设60万吨烯烃、中国化工集团投资140亿建设TDI、ADI、MDI等5个百亿元以上重大转型项目,抓好陕汽5万辆新能源重卡汽车、华润30万吨啤酒、华锐风电装备制造等一批转型项目。继续加大招商引资的力度,加快发展装备制造、新医药、新材料、现代煤化工等高新技术产业,从根本上改变一煤独大的产业格局,率先走出资源型城市转型跨越发展的路子。

我们要以中国大古都引领为龙头,打好大同文化牌。文化是大同的生命和核心竞争力。二十一世纪以文化论输赢。底蕴厚重的历史文化是大同最大的特色和优势。建设中国传统文化标本城市,创造历史文化名城保护的大同模式,是大同文化产业发展的基本战略。下届政府要如期完成云冈大景区深度开发、历史文化名城完整保护、恒山大景区全面提升的目标。以方特欢乐世界为主体的文化创意产业要加快开发,形成与优秀传统文化相映成辉的新兴文化产业支柱。建立文化“大创作”体制,积极利用国内外、省内外文化资源,组织文化大家创作《北魏王朝》电视剧等一批精品力作,精心打造可持续经营、立足大同本土、具有大品牌效应的常态化演出,提高中国古都、天下大同的知名度。推进以弘扬魏碑书法为主体的书法名城建设。加快发展以博物馆为主题的文化会展业,建设中国雕塑馆、中国美术馆大同分馆、中国名人名家收藏馆、世界时装博物馆。持续举办国际雕塑双年展、国际壁画双年展、汽车文化展等各种文化会展活动,不断提升大同文化的重量和厚度,让文化之城、艺术之都、朝圣之地、休闲之区的古都大同更加光彩夺目,打造中国历史文化名城第一品牌,建设世界旅游终极目的地。

我们要以古今并美的双城模式为标志,创造大同特色城。一座饱经千年沧桑的完整古城,延续着曾经的辉煌,传递着历史的回响;一座充满创造活力的现代新区,熔铸着时代的精神,塑造着城市的品格。这就是“一轴双城、古今兼顾”规划思想光照下的大同。大同在历史的迷雾中找到了属于自己的城市坐标,创造出自己的特色个性。文化擦亮城市面孔,现代塑造城市品牌,中国古都,天下大同,一个文化和精神的高地,一个充满文化自信,阔步走向未来的理想城市。下届任期结束时,大同将以独特的个性,在大同的世界里创造大不同,在中国和世界的城市发展史上赢得自己的地位,在全球城市化的浪潮中独树一帜。

三、2012年的主要工作

当前,国际和地区形势复杂多变,国际金融危机深层次影响依然存在,欧债问题前景不明朗,美国就业情况不理想,更凸显了世界经济复苏的曲折性和艰巨性。世界经济环境直接影响到外部需求,国内经济下行的压力加大。基于当前经济形势的判断和把握,我们必须把平稳较快增长放在更加重要的位置。

今年的主要预期目标:全市地区生产总值增长13%,财政总收入增长16%、一般预算收入增长15%,规模以上工业企业增加值增长15%,固定资产投资增长24.5%,社会消费品零售总额增长16%,进出口总额增长5%,城镇居民人均可支配收入增长10%,农民人均纯收入增长14%,城镇新增就业岗位5万个,城镇登记失业率控制在4.2%以内,居民消费价格总水平涨幅控制在4%左右,人口自然增长率控制在7‰以内。

主要约束性指标:万元生产总值综合能耗下降3.5%左右,万元生产总值二氧化碳排放量下降3.7%,二氧化硫、化学需氧量、氨氮排放量分别下降2%、1.3%、1%,氮氧化物排放量与上年持平,烟尘、粉尘排放量均下降3%,万元工业增加值用水量下降4%。

(一)以园区平台为载体,以招商引资为抓手,加大项目落地和开工的力度。

抓住国家转型综改试验区和国际太阳能十项全能竞赛的契机,加快新能源和光伏产业发展。抓好大同协和新能源多晶硅及光伏产业循环经济建设项目,开工建设多晶硅和动力车间,完成太阳能电站一期300MW项目。积极推进台湾耀祥光电、昆仑华阳、天华阳光新能源、阿特斯等太阳能产业项目落地。开工建设中海油、同煤集团40亿立方煤制天然气,同煤60万吨烯烃和10万吨活性炭等重大转型项目。倾全力支持同煤集团实施“双千亿”工程,向世界500强目标快速挺进。确保同煤60万吨甲醇年底投产,为大同转型跨越发展树立标杆。开工建设60万千瓦风电,年底形成120万千瓦风电装机规模。

抓紧已开工和落户签约项目的建设进度,尽快形成现实的经济增长点。5万吨玄武岩连续纤维项目一期、华润20万吨啤酒、陕汽大同新能源专用车、华锐风电装备、中联重科工程机械配套基地等项目今年投产。国药威奇达7-ACA、克拉维酸钾项目达产达效,力争国药集团生物疫苗项目落地。国药阿拉宾度、普德、振东泰盛、同达药业、星宇星火、威奇达光明制药、利群药业、山西仟源、利丰华瑞、惠瑞药业等总投资60亿新建扩产改造项目年内完成。加快推进阳高TDI、ADI项目开工。同煤、天津建材合作投资100亿的灵丘300万吨建材钢项目已破土动工,力争明年竣工投产。

要大力支持和扶植中小微企业的发展,营造全民创业社会环境。积极扶持创新型、创业型、外向型、劳动密集型中小微企业发展。落实政府出台的相关政策,支持普德、新成特炭等中小企业在创业板上市,利用资本市场力量加快中小企业发展。鼓励发展小额贷款和村镇银行,集聚民间资本,化解民营企业融资难题,推动民营经济大发展。

加快兼并重组矿井安全改造和产能提升的进度,完成投资53.8亿元,15座矿井基本建成。积极推进煤电一体化发展战略,充分发挥同煤集团的资源优势,实现同煤与央企各发电大集团强强联合,优势互补,互利共赢。今年同煤2×300MW热电机组竣工投产,力争开工建设二电四期2×1000MW、同煤塔山2×600MW、大唐御东2×300MW等项目。

(二)强化“四轮”联动,继续推进设施农业和重点工程项目,提升县域经济整体水平。

落实扶持和奖励政策,每年设施农业新增3万亩,力争突破5万亩,奖励政策上不封顶。设施农业科技为先导,育苗为基础。今年要建设3个蔬菜集约化、现代化育苗基地、10个部省级蔬菜标准园。抓好福龙生物有限公司蝴蝶兰、木瓜等花卉果蔬连体温室项目,为设施农业树立样板。加快推进标准化健康养殖小区建设,新建、扩建畜牧业标准化养殖园区100个,创建10个国家级标准园区。着力推动“一村一品、一县一业”上规模、提档次、出品牌。抓好雨润、顺鑫两个百万头生猪屠宰及产品深加工、四方高科5000头奶牛项目投产达效,确保承德露露33万吨杏仁露、北京新发地物流园区一期工程10月底投入运营,加快亿丰皮革市场建设。建立覆盖全国的农产品交易平台,扩大农产品销售的市场半径。瞄准京津大市场,规划建设400万头猪、700万只羊、100万头牛的重大养殖项目。引进全国和世界知名品牌龙头企业,是发展大同现代农业的根本,要继续加大力度,实现新的突破。

强化农业基础设施建设。加快实施引黄百万亩灌区、“引黄入左”供水工程。完成唐河水库扫尾工程,开工建设守口堡水库。坚持不懈抓好植树造林工作,牢固树立绿化大同的基本指导思想,以优异成绩迎接全省林业现场会的召开。加强城乡统筹,完成208改线工程,开工建设同浑路新能源园区段一级路17公里改建工程。

抓好新农村建设。完成重点推进村“四化四改”和社会事业发展“五个一”工程,推进新农村集中连片建设,全面完成农村新的“五个全覆盖”。启动六个国家连片特困县片区扶贫开发,完成万人易地扶贫移民,继续加大贫困地区劳动力培训和转移力度。

(三)以中国特色城市为目标,继续推进城市基础设施和园林城市建设,打造乐居幸福的精神家园。

抓好城市道路建设改造。完成45条、110公里的道路建设任务,延伸御东路网框架,完成御西道路改造,实现古城内环贯通,完善园区道路配套,基本建成古城新区四通八达、市民出行方便快捷的路网体系,不断提升城市品位。

加快御东新区开发建设。基本完成太阳宫、博物馆、大剧院、图书馆、美术馆、体育中心等工程主体,开工建设南北长3公里、东西宽600米的文化广场。完成市中级人民法院审判法庭、市公安局110指挥中心大楼等重点工程。推进富力、绿地、恒大、复地、同煤、山煤等大集团在御东开发的速度,加快完成御东中心规划区14个城中村改造,开工建设御东工行大楼、煤运安全生产指挥大楼、新华书店大楼、华建商业文化广场,开工建设大同客运东站,完成机场改扩建工程,初步形成御东新区的基本格局,以现代化城市的靓丽新姿迎接太阳能国际竞赛。

绿色是城市的生命,园林是城市的名片。要全面开展国家园林城市和国家环保模范城市创建工作。完成文瀛湖公园、御河两岸景观绿化、采凉山森林公园、十里河综合公园、口泉植物园等绿化景观工程。实施梓家村森林公园、御河与十里河湿地公园、御东新区公共活动走廊、御东新区西干渠、东小城广场、婚庆公园、南城墙、北城墙带状公园、御东新区东西生态轴等绿化工程,倾力打造绿色宜居新大同,奠定创建国家园林城市的基础。加强环境保护,抓好节能减排,加大源头治理。实施电厂储煤场密闭化改造、粉煤灰综合治理。推进御河、十里河、口泉河、甘河综合治理。实施二电厂和大唐云冈热源改造工程,继续扩大城市集中供热覆盖面。完成御东污水处理厂建设和东、西郊污水处理厂改扩建工程,推进集中供热、供水、供气、垃圾和污水处理全覆盖目标的实现。推进餐厨垃圾处理厂建设工程。坚持公交优先战略,更新公交车200台、出租车700辆,新开通公交线路9条。

提高城市管理水平。继续完善“两级政府、三级管理、四级网络”的管理格局,强化区级政府管理城市的主体功能。深入开展“管理服务年”活动,充分发挥数字化管理平台作用,强化市容环境卫生精细化管理。加强住宅小区特别是政府安居工程物业管理工作,实现环境整洁化、服务人性化、运行有序化、管理标准化。加大市政设施、园林绿化、景区景点维护管理力度,严厉打击偷盗破坏行为。加强宣传教育引导,增强全社会爱我大同的主人翁意识,形成全市人民共同的理想信念、价值追求、伦理习惯和道德规范,提升城市的精神文明水平。

(四)推进名城保护和修复工程,发展现代文化创意产业和文化会展业,实现大同文化的全面复兴。

加快名城保护工程步伐。完成北城墙、西城墙修复工程,基本实现古城城墙四面围合。精心搞好展览馆平移工程,实现古城保护与历史建筑保护的完美统一。实施四牌楼、魁星楼、太平楼、钟楼、鼓楼东西街历史文化街区修复工程,推进代王府和古城东南隅、西南隅里坊四合院修复工程。进一步提升古城保护修复水平和美誉度,充分发挥传统建筑艺术的力量,集中展现砖雕、木雕、石雕艺术,精品荟萃,美轮美奂,再现名城古都的历史辉煌。积极鼓励倡导大同和国内外企业家进军古城,按照古城保护整体规划,参与古城商业、酒店、博物馆和各种文化产业开发,创造古城可持续发展的商业模式。调动全市人民参与文化旅游产业开发的积极性,发挥各种社会团体、企业和个人的能动作用,重视口碑相传,共同打造世界大同的文化品牌。开工云冈窟檐保护工程,全面推进恒山风景名胜大景区建设。

大力发展现代文化创意产业。完成方特欢乐世界主题公园主体工程,形成大品牌领先文化创意产业的格局。建设煤气工业遗址创意产业园、晋华宫矿井文化体验、南城墙时尚灯光秀。建设中国雕塑馆、名人名家个人收藏馆、世界时装馆、中国美术馆大同分馆,丰富和提升大同古城的文化品位,增强古都大同的文化吸引力和影响力。

积极发展大同文化会展业,精心组织第十一届云冈艺术节,举办国际壁画双年展、曾竹韶雕塑艺术奖学金作品展,举办中国大同国际汽车文化节。组织出版天下大同书,策划拍摄电视剧《北魏王朝》,加大大同古都宣传营销的力度。把引进华侨城、港中旅等大集团开发、管理、营销文化产品,作为文化旅游产业大发展的基本途径。积极开发文瀛湖游艇和房车项目,开发大同县温泉、火山群地质公园、生态休闲度假旅游,阳高云林寺、许家窑遗址,天镇慈云寺、新平堡、汉墓群,浑源神溪湿地、汤头温泉、栗毓美墓,灵丘平型关纪念馆、觉山寺、曲回寺,广灵水神堂,左云古长城,新荣得胜堡、方山永固陵等文化旅游产业项目,发展和提升酒店、购物、商贸物流等旅游服务业,加快东小城、云中商城、百盛购物等大型商贸物流商场建设,全面提升五星级酒店管理营销水平,开发富有大同特色的民间工艺文化旅游产品,传承和发展耍孩、罗罗腔、剪纸、绢人、铜器等非物质文化遗产,进一步丰富和深化大同的文化旅游体验。

(五)大力发展各项社会事业,加强和改善民生服务保障体系,夯实人民群众幸福和尊严的基础。

高标准、高质量推进保障性住房建设。城市棚户区改造和保障性住房新开工建设466.56万平米、74561套,续建完工252.42万平米、30160套,确保当年拆迁、当年安置目标的实现。继续推进同煤“两区”建设。加强工程质量监督管理,切实把保障性住房建设成为优质安全的民心工程、德政工程,让人民群众得到真正的实惠。

优先发展教育事业。积极发展学前教育,建设20所标准化幼儿园。大力发展职业教育,完成市高级技校、卫校建设工程,开工建设艺校、体校。新建大同一中初中部,推进大同师范改造建设工程,完成北岳中学、三中、北师大附中、实验小学以及大同四中等15所学校建设工程,推进大同五中、九中、十中、十二中、十三中、铁一中、市特教学校等教育建设工程。

加快医疗卫生事业发展。深化医药卫生体制改革,夯实基层医疗卫生基础,扩大基本药物制度实施范围。加快公立医院改革步伐,控制医药费用的不合理增长。完成市一医院、五医院、中医院、六医院新院建设工程和市二医院搬迁。完成疾控中心综合楼新建工程。开工建设市中心血站。切实加强食品药品监督管理。认真贯彻《全民健身条例》,提高人民群众健康水平。坚持计划生育的基本国策,稳定低生育水平,统筹解决人口问题。

完善社会保障体系。进一步扩大社会保障覆盖范围,全面实施城镇居民基本医疗保险制度。加快破产关闭企业清算终结步伐,确保职工养老、医疗保险落实。做好就业再就业工作,多渠道开辟就业岗位。提高城乡居民最低生活保障标准,切实做到动态管理、应保尽保。推动社会福利事业发展,开工光荣院御东分院二期工程。不断推进殡葬管理改革,完成市殡仪馆新建搬迁工程,迁建马铺山陵园、御祥园树葬公墓。

创新社会管理,完善行政调解和信访工作机制,从源头上预防和化解社会矛盾。完善社会治安防控体系,严密防范和依法打击各种违法犯罪活动。建立健全公共安全管理和突发事件应急体系,有效预防和应对各类突发事件。积极推进安民、便民、惠民的基层社会管理,着力构建人口管理服务体系,大力提升城市现代化管理水平。积极做好民族和宗教工作。巩固双拥成果,加强国防后备力量建设。认真落实安全生产责任制,开展打非治违专项行动,严格执行安全生产的各项法律制度,促进安全生产形势的持续稳定好转。

(六)加强政府自身建设,努力提高执行力和公信力,推进政府职能的根本转变。

建设服务政府,增强为民办事的能力。继续推进政府职能转变,强化政府社会管理和公共服务职能,善于运用创新的思维、改革的思路、务实的方法,解决好发展中的难点问题和人民群众关注的热点问题,提高推动经济发展、促进社会进步、驾驭稳定全局的能力。坚持把务实为民作为政府工作的第一要求,加强对重大决策、重要部署、重点工作的跟踪落实,努力提高政府工作执行力。坚持一切从实际出发,多办顺民意、惠民生、解民忧的实事好事。

建设法治政府,增强依法行政的能力。全面实施“六五”普法规划,把依法行政贯穿于政府决策、执行、监督的全过程,坚持为民行政、为公用权,严格依照法定权限和程序行使权力、履行职责。自觉接受市人大及其常委会的法律监督、工作监督和市政协的民主监督,加强与各民主党派、工商联、人民团体和社会各界的联系,主动接受社会公众监督。坚持政务公开,继续深化行政审批制度改革,确保政府各项工作依法、规范、高效运行。

建设效能政府,增强富民强市的能力。提倡立说立行,即说即办的工作作风,发扬事无巨细、事必躬亲、事不过夜的“三事”精神,落实一线工作法,时时事事掌握调度指挥工作的主动权,以高效创优环境,以环境创造优势,以优势促进发展。

建设廉洁政府,增强防腐拒变的能力。坚持用制度管权、管事、管人,牢记廉洁自律的警戒线、党纪国法的高压线,讲党性、重品行、作表率。做到立身不忘做人之本,为政不移公仆之心,用权不谋一己之私。大兴艰苦奋斗之风,努力节约行政成本,从严控制“三公”费用支出,把公共财政用在保重点、保发展、保民生上,树立勤政、廉政、德政的良好形象。

各位代表,宏伟事业任重道远,美好前景催人奋进。历史和现实的经验证明,我们有目标就能成大事,有坚持就能办实事,有决心就能破难事,有追求就能做好事。让我们紧密团结在以胡锦涛同志为总书记的党中央周围,在省委、省政府和市委的坚强领导下,团结拼搏,励精图治,奋发有为,负重赶超,以转型跨越的优异成绩迎接党的十八大胜利召开!
